# Trends of vital signs with gestational age in normal pregnancies: a systematic review protocol

**DOI:** 10.1136/bmjopen-2015-008769

**Published:** 2016-01-05

**Authors:** Lise Loerup, Rebecca M Pullon, Jacqueline Birks, Susannah Fleming, Lucy H Mackillop, Peter J Watkinson

**Affiliations:** 1 Department of Engineering Science, University of Oxford, Oxford, UK; 2 Centre for Statistics in Medicine, University of Oxford, Oxford, UK; 3 Nuffield Department of Primary Care Health Sciences, University of Oxford, Oxford, UK; 4 Nuffield Department of Obstetrics & Gynaecology, Oxford University Hospitals NHS Trust, Oxford, UK; 5 Nufﬁeld Department of Clinical Neurosciences, Oxford University Hospitals NHS Trust, Oxford, UK

## Abstract

**Introduction:**

Vital signs (blood pressure, heart rate, temperature, oxygen saturation and respiratory rate) are thought to undergo changes during and immediately after pregnancy. However, these physiological changes are not taken into account in the normal ranges, which themselves are not evidence-based, used in routine and acute care monitoring. We aim to synthesise the existing evidence base for changes in vital signs during pregnancy, in order to derive new centile charts for each stage of pregnancy and the immediate postpartum period.

**Methods and analysis:**

We will search the MEDLINE, EMBASE and CINAHL databases from their inception to April 2015 for vital signs from pregnant, intrapartum or postpartum women who were recruited as ‘healthy’. Assessment of bias will be conducted using a predefined set of independently agreed methodological criteria, which assigns an overall quality score to each study. We will record whether the vital sign measurements were made with measurement devices validated for use in pregnancy and in a standard posture. We will use regression methods to construct centile charts of vital signs across pregnancy and the immediate postpartum period for each vital sign. We will compare existing reference ranges to those derived from our centile charts.

**Dissemination:**

The systematic review will be published in a peer-reviewed journal and disseminated electronically and in print.

**PROSPERO reference:**

CRD42014009673.

**Strengths and limitations of this study**

- This is the first review to synthesise the evidence base of vital sign changes during pregnancy, taking into account the gestational age.
- The quality of published information may limit the study findings.
- Combining different methods of measurement of the same vital sign may prove difficult.

## Introduction

### Rationale

Heart rate, blood pressure, respiratory rate, oxygen saturations and temperature are key vital signs used to assess the clinical status of women presenting acutely throughout pregnancy, intrapartum, during anaesthesia and in the early postpartum period. The perceived normal ranges of these vital signs underpin Modified Early Obstetric Warning Scores (MEOWS) developed to assist in early recognition of deterioration.^1^ ^2^ Using these vital signs to detect physiological deterioration is complicated by the normal dynamic changes in maternal vital sign physiology that occur during pregnancy and immediately after delivery. In the case of MEOWS, they define the thresholds that determine if a woman requires review.

Yet currently, normal ranges are either not referenced, or reference a core textbook (which references data from small individual studies published between 1970 and the mid-1990s).^3^ None of the clinical guidelines take account of expected changes in different stages of pregnancy, intrapartum and the early postpartum period. The evidence underpinning current guidance is therefore weak, and thresholds used in clinical practice to detect physiological deterioration appear to be adapted from those established for the non-pregnant population or based on clinical consensus.^1^ ^4^ As apparently small changes in thresholds make substantial differences to the ability of clinical scores to identify physiological deterioration,^5^ ^6^ accurate reference ranges that take into account changes for each stage of pregnancy, the intrapartum and early postpartum periods, are essential to using vital signs to provide high-quality clinical care.

As vital signs are commonly recorded at a particular stage of pregnancy in many different types of clinical studies, large quantities of data may already be available to inform these vital sign thresholds.

### Objectives

We aim to report on existing gestation-specific centiles for vital signs in pregnancy, intrapartum and the early postpartum period using studies of women recruited as ‘healthy’, who undertook vital sign measurements using non-invasive techniques used by healthcare professionals. We will compare the reported centiles with existing reference ranges for each stage of pregnancy, intrapartum and the postpartum period. If the collected data allow, we will attempt to synthesise the reported vital sign data to develop new gestation-specific centile charts.

## Methods

### Registration reference

This systematic review has been registered on PROSPERO (registration number CRD42014009673).

### Criteria for inclusion of studies in this review

#### Types of studies

Prospective and retrospective longitudinal, cross-sectional and case–control studies and randomised control trials will be included.

#### Types of participants

Pregnant women aged 14 years or older, with singleton, normal pregnancies and without illnesses likely to affect the cardiac or respiratory systems will be included.

#### Types of measurements

We will include objective measurement of heart rate, respiratory rate, blood pressure, oxygen saturations or temperature, taken from the start of the antenatal period (early pregnancy) up to 2 weeks post partum. Self-monitoring or other measurements not taken by a healthcare professional, or measurements taken using invasive measurement techniques, will not be included. Gestational age at which the measurements were taken must be reported.

A complete list of inclusion and exclusion criteria has been included in online supplementary appendix table 1, together with a list of acceptable measurement techniques in online supplementary appendix table 2.

#### Types of outcome measures

##### Primary outcome measures

Where centiles are presented, we will report these for each gestational age. Otherwise, where data are not presented as centiles but a sufficient amount of data are available, we will calculate the median and representative centiles (1st, 10th, 25th, 75th, 90th, 99th) for changes in vital signs with respect to gestational age using data from each included study, subject to assessment of normal distributions of vital sign data.

##### Secondary outcome measures

A quality assessment will be performed and a score for the risk of bias for each study will be reported.

Where subgroup analyses suggest that the subgroups have clinically different centile distributions, we will, where sufficient data exist, present subgroup-specific centile distributions.

### Search methods for identifying the studies

#### Electronic searches

Three databases will be searched, from their inception until April 2015: MEDLINE (1950–April 2015), EMBASE (1980–April 2015) and CINAHL (1982–April 2015). Specific search strategies will be developed for each database between clinicians and a qualified librarian from the Oxford University Healthcare Libraries, who will carry out the search. The strategies will use MeSH terms and free text with no language restrictions. An example search strategy is shown in online supplementary appendix table 3.

#### Searching other sources

We will perform non-electronic searches of our own files of articles and of the reference lists of all included studies to identify studies not captured in the initial electronic search.

#### Identification of reference guidelines

PJW and LHM will identify sources of existing reference ranges by reviewing obstetric, physiology and anaesthetic textbooks, international guidelines, standardised clinical training courses and maternal early warning scores to mirror the likely exposure of clinicians to reference ranges.

### Data collection

#### Study selection

**Figure 1:**
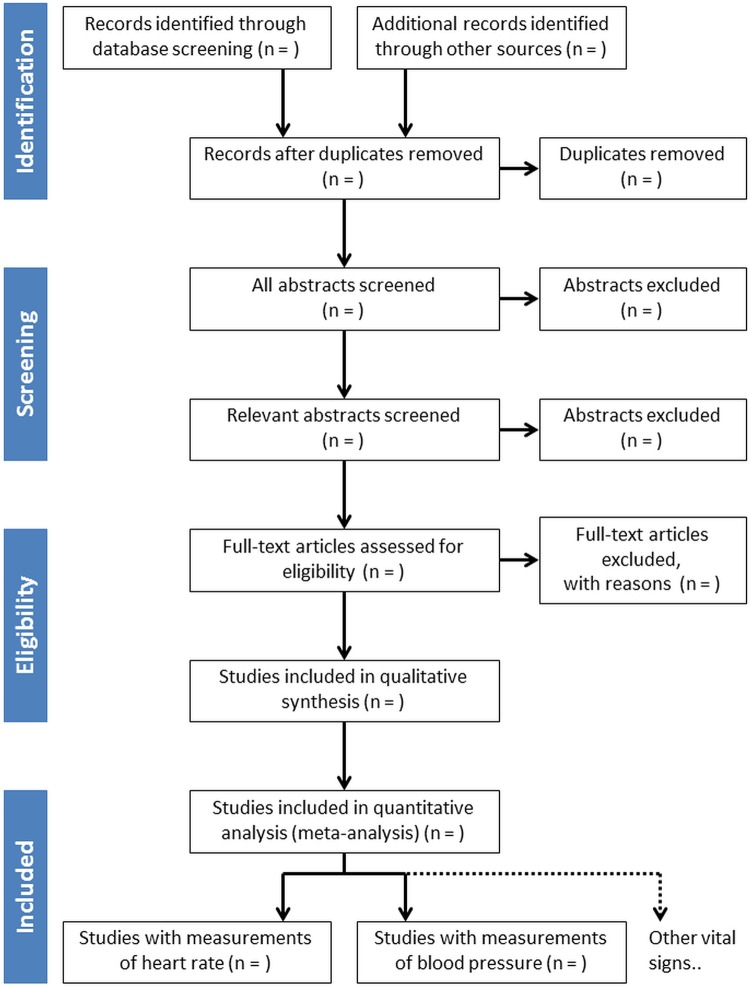
The study selection process, here illustrated by a PRISMA (Preferred Reporting Items for Systematic Reviews and Meta-Analyses) flow diagram.

The retrieved titles and, where available, abstracts will be reviewed by two reviewers (LL and RMP) to exclude studies that clearly fall outside the scope of the review, such as fetal studies or studies not performed on humans. Following this initial sift, the remaining titles and, where available, abstracts will be assessed by two reviewers (PJW and LHM) against the inclusion and exclusion criteria. The full texts of all potentially relevant articles will be retrieved for data extraction where appropriate. Figure 1 shows a PRISMA (Preferred Reporting Items for Systematic Reviews and Meta-Analyses) flow diagram^7^ that summarises the study selection process.

#### Assessment of bias and heterogeneity

A quality assessment of studies that meet our inclusion and exclusion criteria will be performed independently by two reviewers. Disagreements will be resolved by recourse to the original data. The quality assessment will be undertaken in line with the QUADAS-2 assessment,^8^ following the methodology of Ioannou *et al*.^9^ This assessment has been designed to evaluate the methodological quality of observational studies, performed with pregnant women. Where required, the specific assessment criteria will be adapted for our purpose. Results of this quality assessment, which assigns an overall quality score to each study, will be presented in tabular and graphical form.

#### Data extraction and management

Two reviewers will independently perform data extraction (LL and RMP). Data will be extracted into a prepiloted electronic spreadsheet (Microsoft Excel). Disagreements will be resolved by recourse to the original data. Data will be extracted from tables, text or graphs. Appropriate software will be used to ensure accurate transcription of data from graphs, subject to predefined criteria to resolution of graphically presented data as defined in online supplementary appendix table 2. For each period of pregnancy defined in a paper, the number of women in the group will be extracted, along with the following statistical data about the vital signs of interest (blood pressure, heart rate, temperature, oxygen saturation or respiratory rate), where reported:

- Mean value
- Median value
- SD
- Centiles, percentiles, quartiles, etc
- CIs
- SE of the mean.

The period of pregnancy will be extracted as weeks of gestation and the method used to determine gestational age will be recorded. Data for a given period of pregnancy that are reported separately, for example for different ethnic groups or subgroups defined based on a medical diagnosis, will be classified independently. Data from subgroups with a medical diagnosis that could affect their measurement will only be included if the women are described as healthy at the start of the study. When multiple measurements at the same time point are reported for a single physiological variable (eg, lying and sitting heart rates), a single data point will be selected to avoid over-representation using the prespecified rules summarised in online supplementary appendix 3.

In addition, the following data will be extracted from each included paper, if the data are present: date of the study; period of data collection; demographic information about participants (age range, weight, body mass index (BMI), ethnicity, reason for measurements); details of pregnancy (parity, number of gestations); country of study (with subsequent assignment to economic development status, according to the United Nations Development Programme (UNDP) Human Development Index^10^); study setting and details of measurement (subject position, method of measurement, device details).

#### Data extraction from papers of a different language

In order to extract data from studies published in a language other than English, assistance will be sought from people within our research groups (preferably with a medical background) with native proficiency in the relevant language. Data from such studies will be extracted in consultation with one of the two reviewers (LL or RMP).

#### Dealing with missing data

In cases where relevant data have not been adequately reported, or presented in a format that is not suitable for extraction, the original authors will be contacted and the data requested. We will in the first instance use contact details from the original paper, but where these are no longer valid, contact details will be sought from recent publications on PubMed, from institutional websites or through general online search engines. Authors will be contacted twice; initially a request for data will be sent via electronic mail, and if no response is received after 4 weeks, authors will be contacted a second time.

### Data analysis

#### Data synthesis

We will analyse cross-sectional and longitudinal studies separately and pool the vital sign data if appropriate.

##### Cross-sectional studies

Each cross-sectional study will provide a mean response at one or more accurately known gestational age time points. Where a study reports cross-sectional measurements at multiple gestational ages (multiple samples from the same population) the data points will be treated as independent because each participant only contributes one assessment.

Assuming no significant heterogeneity between studies, the centiles and other statistics for each value of gestational age will be reported. If possible, the analysis will pool results from the studies using regression techniques. Where potential confounding factors are reported, such as BMI, malnutrition, and haemoglobin values, we will consider incorporating these factors in a meta-regression. Each study will contribute mean of vital signs and gestational age, taking into account differences in population size. The mean response curve as a function of gestational age will be estimated. If the relationship between the response and gestational age does not appear to have a functional form other non-parametric methods of curve fitting will be used.

##### Longitudinal studies

A longitudinal study measures the response at several time points for each participant. The set of time points may be unique for each participant or identical across participants. The mean response curve over time for each study will be presented graphically, with the equation if a parametric method was used and the equation is reported.

#### Sensitivity analysis

If there is significant heterogeneity at any time point sensitivity analyses will be attempted by dropping outlying studies from the analysis.

#### Subgroup analysis

Where data are available, we will attempt to conduct the following subgroup analyses:

- BMI (or weight) class
- Ethnicity
- Development status of country of study
- Parity
- Position of measurement
- The method of measurement (eg, blood pressure device)
- Measurement setting
- The year of assessment
- Pregnancy complications.

## Discussion

This systematic review will summarise the current state of evidence for trends in maternal physiology in pregnancy. Where sufficient data are available, gestation-specific centile charts of vital signs in pregnancy, intrapartum and the postpartum period will be derived. The knowledge of normal distributions of such data in a low-risk population of women for a *particular stage of pregnancy* is an essential prerequisite both to the development of an evidence-based MEOWS and for best practice use of these vital signs throughout clinical practice.
